# Intestinal obstruction impairs the antitumor function of hepatic natural killer cells against colorectal cancer

**DOI:** 10.1007/s00535-026-02349-w

**Published:** 2026-02-06

**Authors:** Kouki Imaoka, Masahiro Ohira, Takuya Yano, Tomoaki Bekki, Yuki Imaoka, Ryosuke Nakano, Seiichi Shimizu, Manabu Shimomura, Yuka Tanaka, Tsuyoshi Kobayashi, Hideki Ohdan

**Affiliations:** Department of Gastroenterological and Transplant Surgery, Graduate School of Biomedical and Health Sciences, Hiroshima University, 1-2-3 Kasumi, Minami-ku, Hiroshima, 734-8551 Japan

**Keywords:** Intestinal obstruction, Colorectal cancer, Liver metastasis, Tumor necrosis factor-related apoptosis-inducing ligand, Liver natural killer cells

## Abstract

**Background:**

This study aimed to investigate the impact of intestinal obstruction (IO) caused by colorectal cancer (CRC) on the cancer prognosis and recurrence patterns. We analyzed recurrence patterns in patients with stage II–III CRC and employed a murine model to elucidate the effects of IO on hepatic immunity.

**Methods:**

We examined the clinical outcomes of CRC patients with IO and utilized a murine IO model to assess alterations in hepatic immunity, focusing on natural killer (NK) cell function.

**Results:**

IO was significantly associated with poor prognosis and an increased incidence of liver metastases. In the murine model, IO induced hepatic inflammation and impaired the antitumor activity of liver-resident NK cells, whereas its effects on conventional splenic and pulmonary NK cells were minimal. These findings, consistent between human clinical data and murine experiments, suggest that IO promotes a microenvironment conducive to liver metastasis by compromising hepatic immunity.

**Conclusions:**

IO exerts a detrimental effect on hepatic immunity by impairing NK cell-mediated antitumor responses, thereby facilitating liver metastasis in CRC.

**Supplementary Information:**

The online version contains supplementary material available at 10.1007/s00535-026-02349-w.

## Introduction

Obstructive colorectal cancer (CRC) occurs in approximately 8–29% of patients with CRC and accounts for nearly 85% of CRC-related emergencies [1]. This condition is associated with high short-term morbidity and mortality rates [2–4] and may confer a poorer long-term prognosis [5, 6]. Even when tumor-node-metastasis (TNM) stages are matched, patients with obstructive CRC exhibit a higher risk of recurrence than those with non-obstructive CRC. However, the influence of intestinal obstruction (IO) on metastatic patterns remains unclear [7, 8].

The intestinal environment and hepatic antitumor immunity are closely interconnected via the portal circulation [9, 10]. Our previous study demonstrated that liver-resident natural killer (Lr-NK) cells possess distinct phenotypic characteristics and unique antitumor functions compared to peripheral blood NK cells [11]. Lr-NK cells are characterized by tumor necrosis factor-related apoptosis-inducing ligand (TRAIL)^+^ DX5^−^ eomesodermin^−^ T-bet^+^ as an immature population, whereas NK cells at other peripheral sites are predominantly mature in phenotype and function [12, 13]. TRAIL, a member of the TNF superfamily, can initiate the apoptosis pathway by binding to its associated death receptors, which are critical for liver NK cell-mediated anti-tumor cell and antimicrobial killing [14, 15]. As a crucial component of innate immunity, NK cells represent the primary defense mechanism against circulating tumor cells and play a pivotal role in preventing tumor recurrence [16, 17]. Notably, tissue-resident NK cells display phenotypic and functional differences depending on their local microenvironment, which may be key to understanding the mechanisms underlying liver metastasis in patients with obstructive CRC [18].

Tumor-induced colorectal obstruction compromises the gut barrier, allowing microbial components to translocate to the liver via the portal circulation, where they drive sustained hepatic inflammation and may impair antitumor immunity [19]. We hypothesized that this inflammatory response compromises the protective function of liver-resident NK cells against circulating tumor cells, thereby facilitating hepatic metastasis. Elucidating this mechanism may provide valuable insights for the development of clinical strategies to suppress metastasis in patients with obstructive CRC. Therefore, this study aimed to (1) characterize the clinical recurrence patterns of obstructive CRC patients and (2) investigate the effect of IO on the antitumor activity of tissue-resident NK cells using a murine IO model.

## Methods

### Patients

**Fig. 1:**
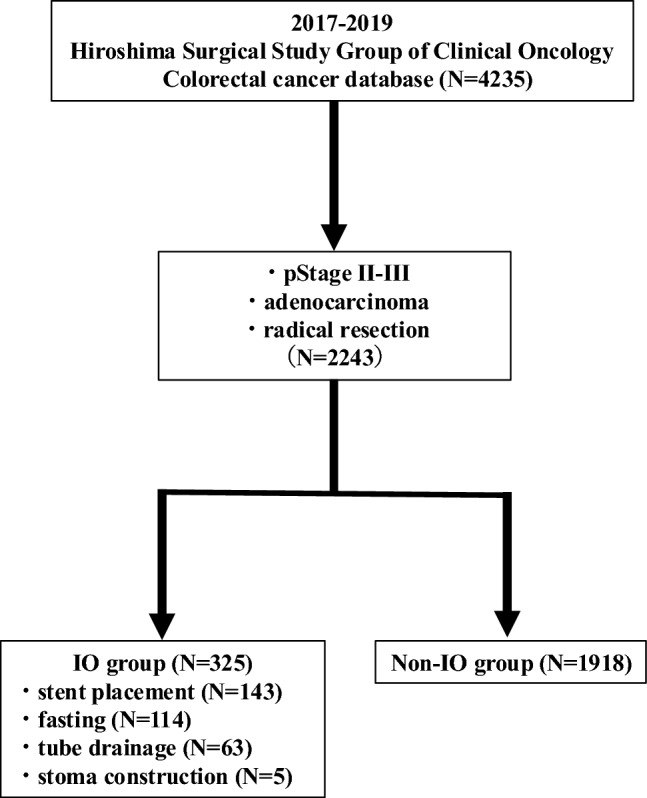
Study consort diagram. Of 4235 patients, 2243 were included in the analysis. The patients were divided into two groups: IO (*n* = 325) and non-IO (*n* = 1918; 1.5%). The patients in the IO group underwent stent placement (*n* = 143), fasting (*n* = 114), tube drainage (*n* = 63), or stoma construction (*n* = 5). IO; intestinal obstruction

This study enrolled patients who underwent curative resection for stage II–III colorectal cancer (CRC) between January 1, 2017, and December 31, 2019, at 15 institutions affiliated with the Hiroshima Surgical Study Group of Clinical Oncology (HiSCO). Patients who underwent surgery for non-malignant diseases or malignancies other than CRC, as well as those with stage I or IV disease or with recurrent CRC, were excluded. Thus, 2243 patients who underwent radical resection for stage II–III disease were enrolled in this study (Fig. 1). The clinical data of the patients, including age, sex, body mass index (BMI), neutrophil-to-lymphocyte ratio (NLR) [20], C-reactive protein/albumin ratio (CAR) [21], Eastern Cooperative Oncology Group performance status (PS), Charlson comorbidity index (CCI) [22], pathological findings, tumor location, and perioperative factors at the time of surgery were extracted. The tumor staging and grading were performed according to the Japanese Society for Cancer of the Colon and Rectum guidelines [23]. IO was diagnosed by the attending physician on the basis of clinical evidence of gastrointestinal symptoms and radiological findings, including colonic dilatation observed on abdominal X-ray or computed tomography. The patients were divided into two groups according to whether they had undergone preoperative therapeutic interventions for IO: an IO (*n* = 325 [14.5%]) group and a non-IO (*n* = 1918 [85.5%]) group. Therapeutic interventions included stent placement (*n* = 143), fasting (*n* = 114), tube drainage (*n* = 63), and stoma construction (*n* = 5).

This study was approved by the Institutional Review Board of Hiroshima University Hospital (approval number E2021-2527). The requirement for obtaining written informed consent was waived owing to the retrospective nature of the study.

### Animal experiments and development of the animal model

Male C57BL/6J (B6) mice aged 8–12 weeks were purchased from CLEA Japan. The mice were housed in the animal facility of Hiroshima University, Hiroshima, Japan, in a pathogen-free microenvironment. This study was performed in strict accordance with the Guide for the Care and Use of Laboratory Animals and the local Committee for Animal Experiments. The anuses of the mice were ligated for five days to create a model of intestinal obstruction (IO). Mice were provided either control or antibiotic (0.5 g/L vancomycin, 1 g/L neomycin sulfate, 1 g/L metronidazole, 1 g/L ampicillin; ABX; Fisher Scientific) containing water for 4 weeks prior to beginning treatment of IO and during IO for 5 days. This ABX regimen has previously been demonstrated to effectively deplete all detectable commensal bacteria [24]. The experimental protocol was approved by the Ethics Review Committee for Animal Experimentation of the Graduate School of Biomedical Sciences, Hiroshima University (Permit Number: A20-97).

### Mouse tumor cell line

The mouse colon adenocarcinoma cell line MC38 (derived from C57BL6 mice) was purchased from Kerafast (USA).

### Flow cytometric analyses

All analytical procedures were performed in accordance with previous studies [21, 25] and can be found in the Supplementary Materials.

### Real-time quantitative reverse transcription polymerase chain reaction (RT-qPCR)

All the analytical procedures are described in the Supplementary Material. The PCR primers used for gene analysis are listed in Table S1.

### Measurement of mouse proinflammatory cytokines via enzyme-linked immunosorbent assay (ELISA)

The Mouse Pro-inflammatory Cytokine Multiplex ELISA Kit was purchased from Arigobio (Hsinchu, Taiwan) and the expression of all serum cytokines was measured according to the manufacturer’s instructions. IL33 concentrations in the serum were determined strictly following the instructions of the enzyme-linked immunosorbent assay (ELISA) kit (R&D Systems, USA). GloMax® Explorer multimode microplate reader (Promega Corporation, USA) was used to measure the absorbance of OPG at 450 nm, with 630 nm as a reference.

### Cytotoxicity assay

Cell cytotoxicity in the mouse model was evaluated as described in previous studies [25, 26] and is described in the Supplementary Materials.

### Statistical analysis

The nonparametric Mann–Whitney U-test was performed to compare differences between the two independent groups; *p*-values < 0.05 were considered statistically significant. Statistical analysis was performed using one-way ANOVA with Tukey post hoc and non-parametric tests (alpha = 0.05). Values were expressed as medians with interquartile ranges (IQR). Overall survival (OS) and recurrence rate (RR) were plotted using Kaplan–Meier analysis and compared using log-rank statistics. Multivariate analyses were conducted for variables that were independently related to each recurrence pattern, using the Cox proportional hazards model. Univariate and multivariate Cox regression analyses were performed to assess the association between the RR and the following variables: age, sex, BMI, PS, CCI, CAR, NLR, CEA, CA19-9, tumor location, poorly differentiated histology, pT4, pN, intestinal obstruction, adjuvant chemotherapy, operation time, and blood loss. All variables were included in the multivariate models, and the backward elimination method with a removal criterion of *p* = 0.05 was used to select covariates. All statistical analyses were performed using the JMP statistical software (JMP® 18; SAS Institute Inc., Cary, NC, USA). Statistical significance was set at *p* < 0.05.

## Results

### Clinicopathological characteristics

The baseline characteristics of the patients in the IO and non-IO groups are presented in Table 1. The CAR and NLR were higher in the IO group than in the non-IO group. The percentage of patients with advanced pT categories was higher in the IO group. No significant differences were found in pN categories or the poorly differentiated pathological histology. Serum CEA and CA19-9 levels were higher in the IO group than in the non-IO group (*p* < 0.001). The rates of postoperative adjuvant chemotherapy did not differ between the two groups.

**Table 1:** Patient characteristics

	Intestinal obstruction (IO) (*N* = 325)	Non-IO (*N* = 1918)	*p* value
Age (years)	74 (65–82)	71 (65–79)	< 0.01
Gender (Male/Female)	168/157	969/949	0.696
BMI (kg/m^2^)	21.2 (18.9–23.7)	22.4 (20.0–24.8)	< 0.01
NLR	3.2 (2.2–4.8)	2.5 (1.9–3.6)	< 0.01
CAR	0.16 (0.05–0.54)	0.03 (0.01–0.14)	< 0.01
CCI			0.359
low (0)	177 (54.5%)	1070 (55.8%)	
medium (1–2)	106 (32.6%)	659 (34.4%)	
high (3–4)	32 (9.9%)	151 (7.9%)	
very high (5–)	10 (3.1%)	38 (2.0%)	
PS			< 0.01
0	192 (59.1%)	1290 (67.3%)	
1	80 (24.6%)	421 (22.0%)	
2	35 (10.8%)	151 (7.9%)	
3	16 (4.9%)	43 (2.2%)	
4	2 (0.6%)	13 (0.7%)	
Tumor location			< 0.01
Colon	256 (78.8%)	1241 (64.7%)	
Rectum	69 (21.2%)	677 (35.3%)	
pT stage			< 0.01
T1	1 (0.3%)	53 (2.8%)	
T2	0 (0.0%)	98 (5.1%)	
T3	199 (61.2%)	1348 (70.5%)	
T4	125 (38.5%)	414 (21.6%)	
pN stage			0.571
N0	165 (50.8%)	977 (51.0%)	
N1	108 (33.2%)	684 (35.7%)	
N2	43 (13.2%)	214 (11.2%)	
N3	9 (2.8%)	41 (2.1%)	
Por/muc (+)	42 (13.0%)	214 (11.2%)	0.353
CEA (ng/mL)	5.9 (3.3–12.7)	3.9 (2.2–8.8)	< 0.01
CA19-9 (U/mL)	13.2 (6.0–24.1)	9.4 (4.2–20.1)	< 0.01
Adjuvant chemotherapy (+)	131 (40.4%)	857 (45.2%)	0.107
Operation time (min)	234 (189–306)	228 (183–287)	0.222
Blood loss (mL)	53 (20–186)	40 (13–100)	< 0.01

*BMI* body mass index, *NLR* neutrophil-to-lymphocyte ratio, *CAR* C-reactive protein/albumin ratio, *CCI* Charlson comorbidity index, *PS* performance status, *pT* pathological tumor, *pN* pathological lymph node, *Por/muc* poorly/mucinous, *CEA* carcinoembryonic antigen, *CA19-9* carbohydrate antigen 19-9

### Kaplan–Meier survival curve analysis between the IO and non-IO groups

**Fig. 2:**
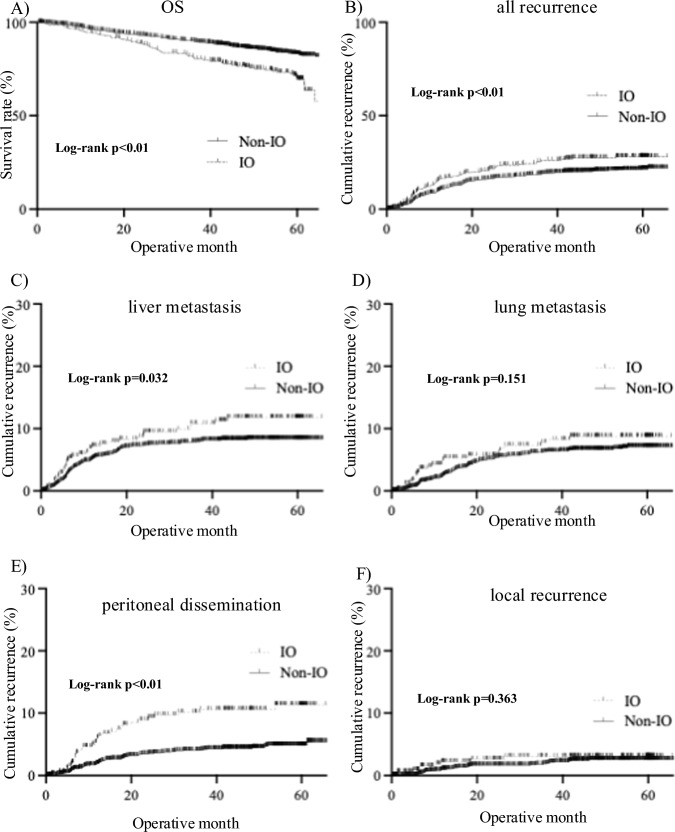
Long-term prognosis and recurrence patterns. **A** Kaplan–Meier survival curve analysis showed worse overall survival rates in the IO group than in the non-IO group. **B** Kaplan–Meier survival curve analysis showed worse recurrence rates in the IO group compared with the non-IO group. **C** Kaplan–Meier survival curve analysis showed worse liver metastasis in the IO group compared to the non-IO group. **D** Kaplan–Meier survival curve analysis showed no significant difference in lung metastasis between the two groups. **E** Kaplan–Meier survival curve analysis showed worse peritoneal dissemination in the IO group compared to the non-IO group. **F** Kaplan–Meier survival curve analysis showed no significant difference in local recurrence between the two groups

The median follow-up time from radical surgery was 49.1 months (IQR, 38.7–59.8 months) for the IO group and 47.8 months (IQR, 25.6–59.5 months) for the non-IO group. The 5-year OS rates in the IO and non-IO groups were 70.6% and 83.0%, respectively (*p* < 0.01; Fig. 2A); whereas the 5-year RR in the IO and non-IO groups were 30.9% and 21.5%, respectively (*p* < 0.01; Fig. 2B). The primary sites of recurrence from the overall cohort were liver metastases (*n* = 173, 7.7%), lung metastases (*n* = 132, 5.9%), peritoneal dissemination (*n* = 104, 4.6%), and local recurrence (*n* = 46, 2.1%). We compared the cumulative incidence of each recurrence pattern between the IO group and the non-IO group to evaluate whether IO was associated with a higher risk of recurrence at specific metastatic sites. Kaplan–Meier survival curve analysis showed significantly worse liver metastasis and peritoneal dissemination in the IO group than in the non-IO group, while there was no significant difference in lung metastasis and local recurrence between the two groups (Fig. 2C–F). Next, we examined the effect of each preoperative intervention for IO on liver metastasis as a subgroup analysis. There was no statistically significant difference in the recurrence rate of liver metastasis between each preoperative treatment intervention group and the non-IO group (Supplemental Fig. 1).

### Factors associated with each recurrence patterns

Multivariate analysis identified obstruction (hazard ratio [HR], 1.47; 95% confidence interval [CI], 1.16–2.57; *p* < 0.01), rectal cancer (HR, 1.46; 95% CI, 1.19–1.79, *p* < 0.01), pT4 stage (HR, 1.86; 95% CI, 1.52–2.28, *p* < 0.01), pN stage (pN1: HR, 2.04; 95% CI, 1.62–2.57, *p* < 0.01; pN2: HR, 3.41; 95% CI, 2.57–4.52, *p* < 0.01; pN3: HR, 4.35; 95% CI, 2.75–6.90, *p* < 0.01), absence of adjuvant chemotherapy (HR, 1.97; 95% CI, 1.41–2.73, *p* < 0.01), and operation time (HR, 1.00; 95% CI, 1.00–1.00, *p* < 0.01) as independent predictive factors for all recurrence (Table 2). A sub-analysis of recurrence patterns indicated that obstruction was not an independent risk factor for lung metastasis (Table S2) or local recurrence (Table S4), but it was for liver metastasis (Table 3) and peritoneal dissemination (Table S3).

**Table 2:** Risk factors for all recurrence

Factors	Univariate			Multivariate		
	HR	95% CI	p-value	HR	95% CI	*p*-value
Age, per 1 year	1.00	1.00–1.01	0.388			
Gender (Male)	0.96	0.79–1.15	0.636			
BMI (kg/m^2^), per 1 kg/m^2^	0.98	0.96–1.01	0.206			
PS						
0	1					
1	1.25	1.00–1.56	0.052			
2	1.63	1.18–2.24	0.003			
3	1.94	1.12–3.39	0.020			
4	0.61	0.09–4.37	0.614			
CCI, per 1	1.07	1.01–1.15	0.033			
CAR, per 1	1.04	0.96–1.10	0.312			
NLR, per 1	1.03	1.00–1.05	0.040			
CEA, per 1 ng/mL	1.00	1.00–1.00	0.009			
CA19-9, per 1 U/mL	1.00	1.00–1.00	0.118			
Tumor location (rectum)	1.48	1.23–1.79	< 0.01	1.46	1.19–1.79	< 0.01
Histological type (Por/muc)	1.33	1.02–1.74	0.036			
pT4	2.17	1.79–2.62	< 0.01	1.86	1.52–2.28	< 0.01
pN						
N0	1			1		
N1	1.74	1.40–2.16	< 0.01	2.04	1.62–2.57	< 0.01
N2	2.98	2.29–3.86	< 0.01	3.41	2.57–4.52	< 0.01
N3	4.53	2.95–6.97	< 0.01	4.35	2.75–6.90	< 0.01
Obstruction (+)	1.58	1.25–1.99	< 0.01	1.47	1.16–1.87	< 0.01
No adjuvant chemotherapy	1.12	0.93–1.36	0.221	1.97	1.41–2.73	< 0.01
Operation time, per 1 min	1.00	1.00–1.00	< 0.01	1.00	1.00–1.00	< 0.01
Blood loss, per 1 mL	1.00	1.00–1.00	< 0.01			

*BMI* body mass index, *NLR* neutrophil-to-lymphocyte ratio, *CAR* C-reactive protein/albumin ratio, *CCI* Charlson comorbidity index, *PS* performance status, *pT* pathological tumor, *pN* pathological lymph node, *Por/muc* poorly/mucinous, *CEA* carcinoembryonic antigen, *CA19-9* carbohydrate antigen 19-9

**Table 3:** Risk factors for liver metastasis

Factors	Univariate			Multivariate		
	HR	95% CI	p-value	HR	95% CI	*p*-value
Age, per 1 year	1.00	0.99–1.02	0.770			
Gender (Male)	1.23	0.91–1.66	0.179			
BMI (kg/m^2^), per 1 kg/m^2^	0.99	0.95–1.03	0.685			
PS						
0	1					
1	1.22	0.85–1.75	0.277			
2	1.71	1.03–2.81	0.036			
3	1.81	0.74–4.44	0.194			
4	1.45	0.20–10.4	0.711			
CCI, per 1	1.11	1.00–1.22	0.044			
CAR, per 1	0.92	0.76–1.06	0.289			
NLR, per 1	0.99	0.94–1.03	0.771			
CEA, per 1 ng/mL	1.00	1.00–1.00	0.205			
CA19-9, per 1 U/mL	1.00	1.00–1.00	0.263			
Tumor location (rectum)	1.26	0.93–1.71	0.147			
Histological type (Por/muc)	1.07	0.67–1.71	0.764			
pT4	1.45	1.05–2.02	0.026			
pN						
N0	1			1		
N1	2.18	1.55–3.07	< 0.01	2.89	2.01–4.16	< 0.01
N2	2.65	1.69–4.13	< 0.01	3.77	2.35–6.05	< 0.01
N3	3.06	1.32–7.12	< 0.01	4.60	1.94–10.9	< 0.01
Obstruction (+)	1.51	1.03–2.21	0.041	1.47	1.00–2.15	0.048
No adjuvant chemotherapy	1.25	0.92–1.69	0.151	1.97	1.41–2.73	< 0.01
Operation time, per 1 min	1.00	1.00–1.00	0.821			
Blood loss, per 1 mL	1.00	1.00–1.00	0.134			

*BMI* body mass index, *NLR* neutrophil-to-lymphocyte ratio, *CAR* C-reactive protein/albumin ratio, *CCI* Charlson comorbidity index, *PS* performance status, *pT* pathological tumor, *pN* pathological lymph node, *Por/muc* poorly/mucinous, *CEA* carcinoembryonic antigen, *CA19-9* carbohydrate antigen 19-9

### Correlation between intestinal obstruction and the function of liver NK cells in mice model

**Fig. 3:**
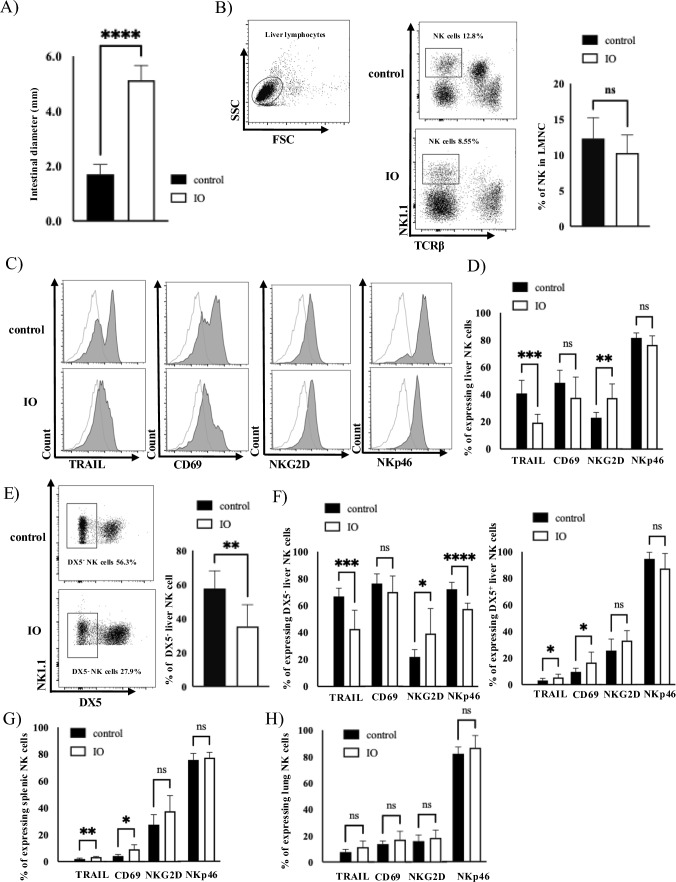

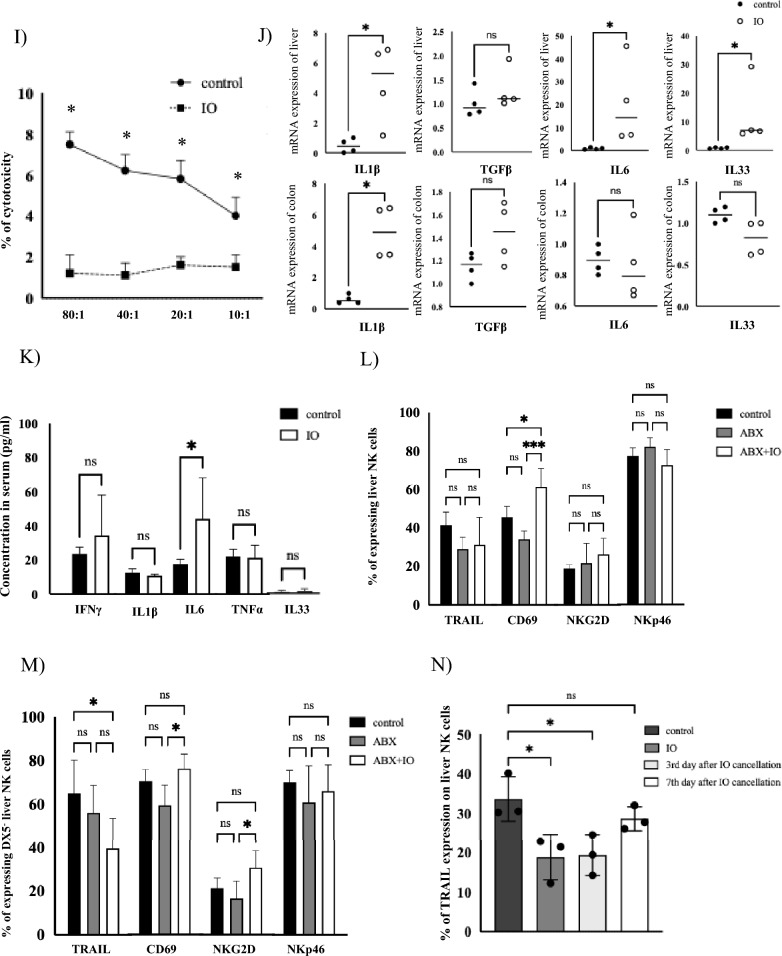
Correlation between intestinal obstruction and the function of liver NK cells in mice model. **A** Intestinal diameter was significantly larger in IO mice (5.2 ± 0.6 mm, *n* = 4) compared to control mice (1.7 ± 0.4 mm, *n* = 4). **B** Samples from control mice (upper panel) and IO mice (lower panel). The proportion of liver NK cells identified as NK1.1^+^TCRβ^−^ liver NK cells in both mice (control (*n* = 8) and IO mice (*n* = 8)). The population of liver NK cells in the control mice was 12.3 ± 2.9%, while that of liver NK cells in IO mice was 10.3 ± 2.5%, respectively. **C** Histograms show activation markers specifically gated on NK1.1^+^TCRβ^−^ liver NK cells. Transparent graphs correspond to isotype controls. Representative histograms of TRAIL, CD69, NKG2D, and NKp46 expression in liver NK cells from control (upper) and IO (lower) mice. **D** The proportions of TRAIL-, CD69-, NKG2D- and NKp46-positive cells among NK1.1^+^TCRβ^−^ NK cells are shown in both control mice (*n* = 8) and IO mice (*n* = 8). Flow cytometric analysis revealed that TRAIL, CD69, NKG2D, and NKp46 expressions in liver NK cells of the control mice were 40.8 ± 9.6%, 48.6 ± 9.2%, 22.9 ± 3.9%, and 81.8 ± 3.6%, while those of IO mice were 19.3 ± 6.1%, 37.5 ± 15.3%, 37.4 ± 10.4%, and 76.5 ± 6.8%, respectively. **E** Proportions of DX5^−^ and DX5^+^ liver NK cells in control (upper) and IO (lower) mice. The proportion of DX5^−^ liver NK cells in control mice was 57.5 ± 10.5%, while that of DX5^−^ liver NK cells in IO mice was 35.3 ± 12.9%, respectively. **F** Proportions of TRAIL-, CD69-, NKG2D- and NKp46-positive cells among DX5^−^ and DX5^+^ liver NK cells are shown in both mice. Flow cytometric analysis revealed that TRAIL, CD69, NKG2D, and NKp46 expressions in DX5⁻ liver NK cells of control mice were 66.8 ± 6.1%, 76.4 ± 7.1%, 22.0 ± 5.4%, and 72.1 ± 5.2%, while those of IO mice were 42.6 ± 14.0%, 70.0 ± 11.9%, 39.2 ± 18.7%, and 57.6 ± 4.2%, respectively. The corresponding expressions in DX5⁺ liver NK cells were 3.1 ± 1.6%, 9.7 ± 2.7%, 25.5 ± 8.8%, and 94.7 ± 4.9% in control mice, and 5.4 ± 2.5%, 16.6 ± 7.9%, 33.1 ± 7.5%, and 87.3 ± 11.4% in IO mice, respectively. G) Proportions of TRAIL-, CD69-, NKG2D- and NKp46-positive cells among splenic NK cells are shown in both mice groups. Flow cytometric analysis revealed that TRAIL, CD69, NKG2D, and NKp46 expressions in splenic NK cells of control mice were 2.0 ± 0.7%, 4.2 ± 1.1%, 27.6 ± 7.5%, and 75.5 ± 4.9%, while those of IO mice were 3.3 ± 0.6%, 9.1 ± 3.4%, 37.5 ± 11.6%, and 77.2 ± 3.9%, respectively. **H** The proportions of TRAIL-, CD69-, NKG2D- and NKp46-positive cells among lung NK cells are shown in both mice groups. Flow cytometric analysis revealed that TRAIL, CD69, NKG2D, and NKp46 expressions in lung NK cells of control mice were 7.5 ± 2.1%, 13.6 ± 2.4%, 15.9 ± 4.7%, and 82.1 ± 5.1%, while those of IO mice were 12.5 ± 4.6%, 17.0 ± 4.6%, 20.0 ± 6.5%, and 86.7 ± 9.3%, respectively. **I** Cytotoxicity of LMNCs against MC38 cell lines in control and IO mice. Data are expressed as mean ± SD (3 mice per group), **J** Difference in mRNA expression of proinflammatory cytokines such as IL1β, IL6, and TGFβ in the liver and colon among mice (four mice per group) was analyzed using quantitative real-time polymerase chain reaction (q-PCR). **K** Comparison of proinflammatory cytokines, such as IFNγ, IL1β, IL6, TNFα and IL33, in the portal vein between control and IO mice (*n* = 4 per group). **L** Changes of TRAIL expression on liver NK cells after canceling IO. Statistical differences were detected using an unpaired t-test or Wilcoxon test, when appropriate. *p < 0.05; **p < 0.01; ***p < 0.001; ****p < 0.0001

Owing to the difficulty of retrospectively obtaining liver NK cells from clinical liver specimens of patients with CRC treated with IO, a detailed investigation was conducted using an IO mouse model. The average intestinal diameter was larger in the IO mice than in the control mice (Fig. 3A). There was no significant difference in the proportion of liver NK cells between the control and IO mice (Fig. 3B). Figure 3C shows representative histograms of tumor necrosis factor-related apoptosis-inducing ligand (TRAIL), CD69, NK group 2 member D (NKG2D), and NKp46 expression in the liver NK cells in both control and IO mice. The expressions of activation markers, including TRAIL and NKp46, on NK cells were significantly decreased, whereas NKG2D expression was significantly increased in IO mice. A decrease in the proportion of CD49b (DX5)^−^NK cells (defined as liver-resident NK cells; lr-NK) was observed in the IO mice (Fig. 3D). In addition, TRAIL and NKp46 expression in lr-NK cells was remarkably decreased in IO mice compared to that in control mice, whereas TRAIL and CD69 expression in DX5^+^ liver NK cells and splenic NK cells was slightly increased in IO mice compared to that in control mice (Fig. 3F, G). No significant difference was observed in the markers of lung NK cells between the control and IO mice (Fig. 3H). Furthermore, the cytotoxicity of liver mononuclear cells (LMNCs) against colon cancer cells (MC38) was lower in IO mice than in control mice (Fig. 3I). Analysis of changes in inflammatory and pro-fibrotic cytokines revealed that mRNA expression of IL-6, IL-1β and IL-33 in the liver was increased in IO mice compared to that in control mice, while IL-1β mRNA expression in the colon was increased in IO mice compared to that in control mice (Fig. 3J). The mean serum IL-6 level in the portal vein was higher in the IO mice than in the control mice (Fig. 3K). We have previously reported that IL‐33 co‐culture significantly suppressed TRAIL expression on liver NK cells [26]. To confirm the functional role of other cytokines, such as IL‐1β and IL‐6, in liver NK cells, LMNCs were cultured in the presence of recombinant IL-6 or IL-1β (0, 10, and 100 ng/mL) for 4 h, but the percentage of TRAIL expression in liver NK cell remained unchanged (Supplemental Fig. 2A, B). After depletion of the gut microbiota with ABX-containing water, we assessed the impact of IO on hepatic NK cell responses. Notably, antibiotic treatment preserved TRAIL expression on both liver NK cells and lr-NK cells following IO (Fig. 3L, M). Seven days after IO discontinuation, TRAIL expression in liver NK cells improved compared to that in control mice (Fig. 3N).

## Discussion

In this study, a multicenter retrospective analysis demonstrated that preoperative IO in patients with CRC was associated with a poorer OS and a higher RR, particularly in relation to liver metastasis and peritoneal dissemination. To the best of our knowledge, few studies have specifically reported the negative effect of preoperative IO on liver metastases in CRC. Additionally, findings from a mouse model revealed that although IO exerts minimal influence on the function of peripheral blood NK cells, it significantly impairs the antitumor activity of lr-NK cells and induces inflammatory alterations in the hepatic microenvironment via the portal vein. These results suggest that mitigating inflammatory changes in patients with obstructive CRC may help reduce the risk of liver metastasis by preserving the hepatic antitumor immunity.

Previous studies have yielded inconsistent findings regarding the effects of IO on CRC recurrence patterns. Some studies have suggested that IO is primarily associated with systemic recurrence rather than peritoneal or local recurrence in patients with stage III disease [5]. In contrast, Cortet et al. reported that IO is an independent risk factor for local recurrence and is significantly associated with distant metastases [7]. Another study reported a higher incidence of synchronous peritoneal metastasis in patients with IO than in those without IO [8]. However, because most of these studies were conducted at single centers, their results are limited in generalizability. In contrast, our large-scale multicenter analysis indicated that IO was significantly associated with liver metastasis and peritoneal dissemination but not with lung metastasis or local recurrence.

Recent research has increasingly emphasized the critical influence of the intestinal environment on hepatic immunity mediated through the portal venous system. The liver, which is rich in innate immune cells, plays a central role in immune regulation and is constantly exposed to circulating antigens and endotoxins from the gut microbiota [27]. Gut microbes and their metabolites entering the portal vein significantly influence liver physiology and immune homeostasis. Previous studies have shown that gut dysbiosis can regulate the liver immune environment or the immune response of lr-NK cells through several mechanisms, including the activation of intestinal inflammation and the accumulation of bacterial and metabolic products [28–33]. In patients with IO, the integrity of the intestinal mucosal barrier is compromised, resulting in dysbiosis and an imbalance in the intestinal microbiota. This disruption permits the translocation of bacteria and endotoxins into the portal circulation, exacerbating hepatic inflammation and further impairing liver function [34, 35]. In patients with CRC, IO has been shown to further alter the gut microbiota composition, increasing microbial diversity and weakening the intestinal barrier. These changes can damage the mucus layer and epithelial lining, triggering immune responses that promote chronic inflammation and contribute to poor clinical outcomes [36, 37]. Our findings support this hypothesis. In the IO mouse model, we observed significantly elevated mRNA expression of key inflammatory cytokines in the liver and increased serum cytokine levels in the portal venous blood. IL-33 is a nuclear cytokine of the IL-1 family that emerged as a critical modulator in inflammatory disorders. Our previous study indicated that IL-33 directly decreased TRAIL expression in lr-NK cells via AKT-forkhead box O and mitogen-activated protein kinase signaling [26]. In the present study, our findings indicate that IO induces upregulation of IL-33 in the liver. Furthermore, after depleting all detectable commensal bacteria, IO did not affect the TRAIL expression on lr-NK cells. This might provide us a clue suggesting dysbiosis and an imbalance in the intestinal microbiota could have caused suppression of lr-NK activity. However, in our retrospective clinical cohort, detailed information regarding antibiotic administration or probiotic use during the IO period was not available, precluding direct clinical validation of this hypothesis. Multiple complex mechanisms may jointly regulate hepatic immune responses during IO, and further prospective studies integrating perioperative microbiota status, immune function, and metastatic outcomes will be required to fully elucidate these pathways.

Developing strategies to overcome TRAIL resistance is expected to advance TRAIL-based therapies with significant potential for widespread application in the treatment of human cancers, including CRC [38]. IO had little effect on the activation of lung NK cells, splenic NK cells, or DX5^+^liver NK cells, but significantly suppressed TRAIL expression on lr-NK cells and significantly reduced the cytotoxic activity of LMNCs. Notably, even after the resolution of IO, TRAIL expression in lr-NK cells required up to 7 days to return to baseline levels. While this timeframe cannot be directly extrapolated to humans, it suggests the existence of an immunologically vulnerable period following decompression during which hepatic immune surveillance may remain compromised. From a clinical perspective, this perioperative window may be associated with increased exposure to circulating tumor cells and heightened susceptibility to tumor engraftment, thereby facilitating the establishment of micrometastases in the liver.

This study had some limitations that should be considered when interpreting its findings. First, this was a retrospective, non-randomized cohort study, which may have resulted in potential selection and detection biases. Second, the referenced databases did not contain information on the RAS and RAF gene mutation status in the tumors. Additionally, T cells, which play a central role in the immune response against solid tumors, were not evaluated. Furthermore, our mouse model utilized a mechanically induced IO rather than a tumor-related obstruction. This simplified system allowed us to focus on the direct biological effects of obstruction, independent of tumor-derived factors. However, malignant obstruction is accompanied by additional pathological elements, including tumor progression, cancer-associated inflammation, and tumor–immune interactions, which are not fully recapitulated in this model. Therefore, further studies incorporating colorectal cancer–induced obstruction models are warranted to more accurately reflect the clinical condition. In addition, although no mortality or overt signs of severe illness were observed in the 5-day anal ligation model, detailed longitudinal assessments of systemic condition—such as body-weight changes, hydration status, and food intake—were not systematically quantified. Therefore, we cannot completely exclude the possibility that some immune alterations reflect nonspecific systemic stress associated with prolonged obstruction. Future studies incorporating standardized clinical scoring and physiological monitoring will be important to further validate obstruction-specific immune effects.

In conclusion, this study clarified that preoperative IO has a negative impact on cancer recurrence, especially liver metastasis, after the radical resection of stage II–III CRC. IO may suppress the antitumor activity of lr-NK cells, leading to the progression of liver metastasis.

## Supplementary Information

Below is the link to the electronic supplementary material.

Supplementary file1 (DOCX 37 KB)

Supplementary file2 (PPTX 205 KB)

Supplementary file3 Supplemental Table 1. Risk factors for lung metastasis. Supplemental Table 2. Risk factors for peritoneal dissemination. Supplemental Table 3. Risk factors for local recurrence (DOCX 57 KB)
