# Supplementary material for: Intestinal obstruction impairs the antitumor function of hepatic natural killer cells against colorectal cancer
Source: J Gastroenterol. 2026 Feb 6;61(4):450–61. doi: 10.1007/s00535-026-02349-w (PMC13048930; doi:10.1007/s00535-026-02349-w)
Supplement: Supplementary file 1 — Supplementary file1 (DOCX 37 KB) [file 535_2026_2349_MOESM1_ESM.docx]

**Supplementary materials**

**Flow cytometric analysis**

Flow cytometric analysis was performed using a FACS Canto II (BD Biosciences, Mountain View, CA, USA). Freshly isolated mononuclear cells from the mouse model were preincubated with anti-CD16/32 (2.4G2) monoclonal antibody to block nonspecific FcγRII/III binding, followed by staining with fluorescently conjugated monoclonal antibodies. To characterize the phenotype of NK cell surface markers, liver leukocytes were stained with the subsequent mAbs: anti-NK1.1 (PK136), anti-CD69 (H1.2F3), anti-DX5 (CD49b), anti-NKG2D (CD314), and anti-NKp46 (CD335) - all procured from BD Pharmingen, San Diego, CA, USA - along with anti-TCRβ chain and anti-TNF-related apoptosis-inducing ligand (TRAIL) (CD253) sourced from BioLegend, San Diego, CA, USA. Dead cells, identified via forward scatter and propidium iodide (PI; Sigma-Aldrich, St. Louis, MO, USA), along with staining, were omitted from the analysis as per previous reports (1, 2).

**Quantitative RT-PCR**

Total RNA was extracted by the RNeasy Mini Kit (Qiagen, Venlo, The Netherlands), and cDNA was synthesized using the Quanti Tect Reverse Transcript Kit (Qiagen). PCRs were performed using SYBR Green PCR Master Mix (Applied Biosystems, Foster City, CA, USA). Polymerase chain reactions (PCRs) were performed in triplicate with 20 μL reaction mixtures using SYBR Green PCR Master Mix (Applied Biosystems, Foster City, CA, USA), following the manufacturer's instructions. Gene expression levels were measured using a Rotor-GeneQ (Qiagen, Hilden, Germany). The amplification protocol consisted of denaturation at 95˚C for 5 min, followed by 40 cycles of 95˚C for 5 s and 60˚C for 10 s. Relative quantities were calculated using the ΔΔCt formula and normalized against the transcript levels of the housekeeping gene β-2 macroglobulin as in the previous study protocol. The PCR primers used for the gene analysis are shown in Supplemental Table 1.

**Cytotoxicity assay**

　Target cells lines were labeled with Sodium Chromate (Na2 [51Cr] O4) and subsequently incubated with effector cells in round-bottomed 96-well plates at 37˚C for a duration of 4 hours. Control cells comprised target cells incubated either in the culture medium to determine spontaneous release, or in a 2% Nonidet P-40 mixture to ascertain maximum 51Cr release. The percentage of cytotoxicity, inferred from the 51Cr release, was computed using the following formula:

Percent cytotoxicity = [(CPM of experimental release - CPM of spontaneous release)] / [(CPM of maximum release - CPM of spontaneous release)] × 100

This was measured utilizing a gamma counter (Aloka ARC-380).

**Isolation of mouse lymphocytes**

Liver lymphocytes were isolated according to a previously described protocol (1). In brief, 1 mL of phosphate-buffered saline (PBS) containing 10% heparin was injected via the portal vein, followed by liver perfusion with 50 mL of PBS supplemented with 0.1% ethylenediaminetetraacetic acid (EDTA). Perfusate-derived cells were collected by centrifugation, and erythrocytes were removed using ammonium-chloride-potassium (ACK) lysis buffer.

Reference

1. Yano T, Ohira M, Nakano R, Tanaka Y, Ohdan H. Hepatectomy leads to loss of TRAIL-expressing liver NK cells via downregulation of the CXCL9-CXCR3 axis in mice. PLoS ONE. 2017; 12(10):e0186997.
2. Ohira M, Hotta R, Tanaka Y, Matsuura T, Tekin A, Selvaggi G, et al. Pilot study to determine the safety and feasibility of deceased donor liver natural killer cell infusion to liver transplant recipients with hepatocellular carcinoma. Cancer Immunol Immunother 2022;71:589–99.
