# Supplementary material for: Intestinal obstruction impairs the antitumor function of hepatic natural killer cells against colorectal cancer
Source: J Gastroenterol. 2026 Feb 6;61(4):450–61. doi: 10.1007/s00535-026-02349-w (PMC13048930; doi:10.1007/s00535-026-02349-w)
Supplement: Supplementary file 2 — Supplementary file2 (PPTX 205 KB) [file 535_2026_2349_MOESM2_ESM.pptx]

## Slide 1
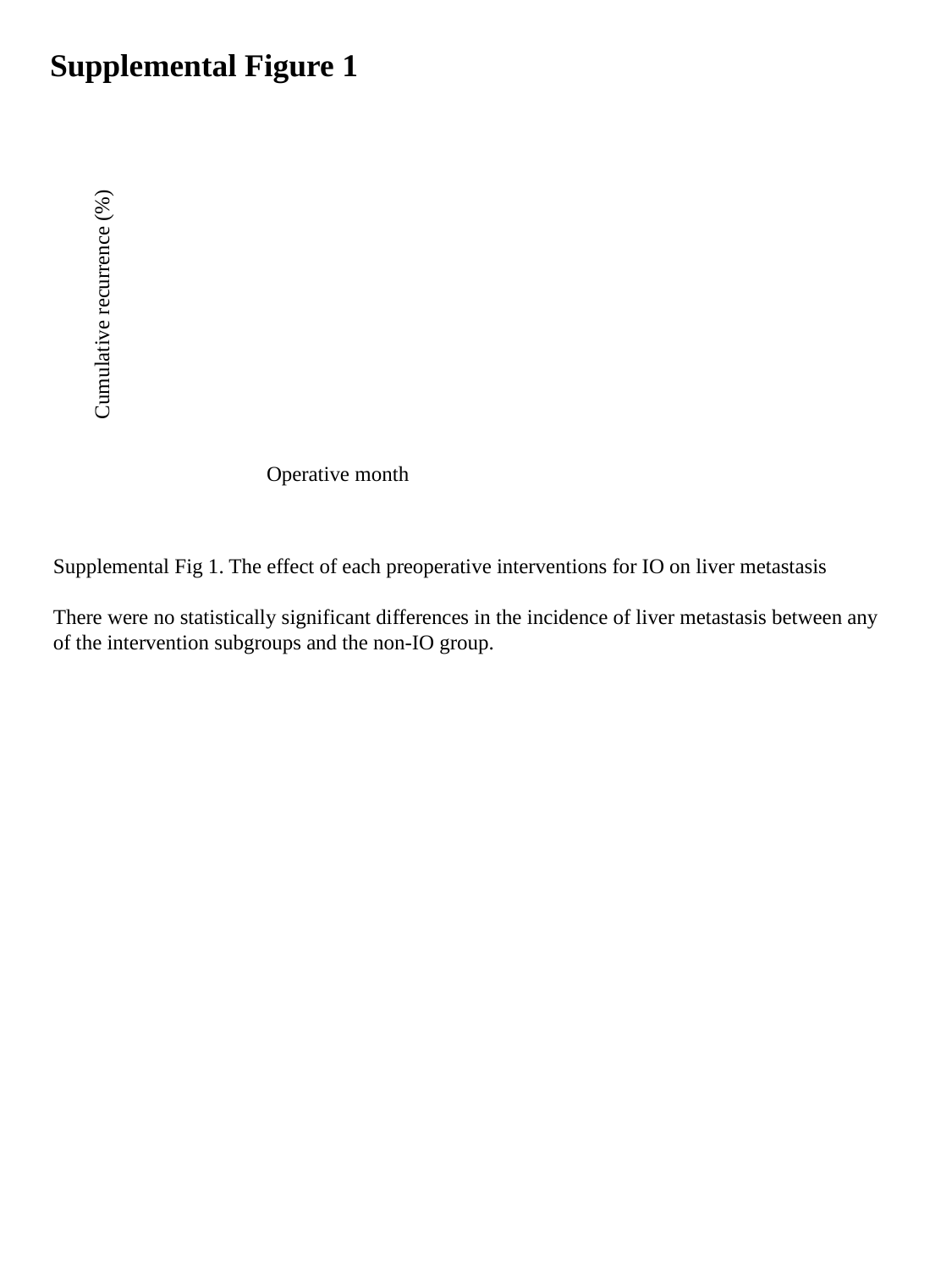

Supplemental Figure 1
Cumulative recurrence (%)
Operative month
Supplemental Fig 1. The effect of each preoperative interventions for IO on liver metastasis
There were no statistically significant differences in the incidence of liver metastasis between any of the intervention subgroups and the non-IO group.

## Slide 2
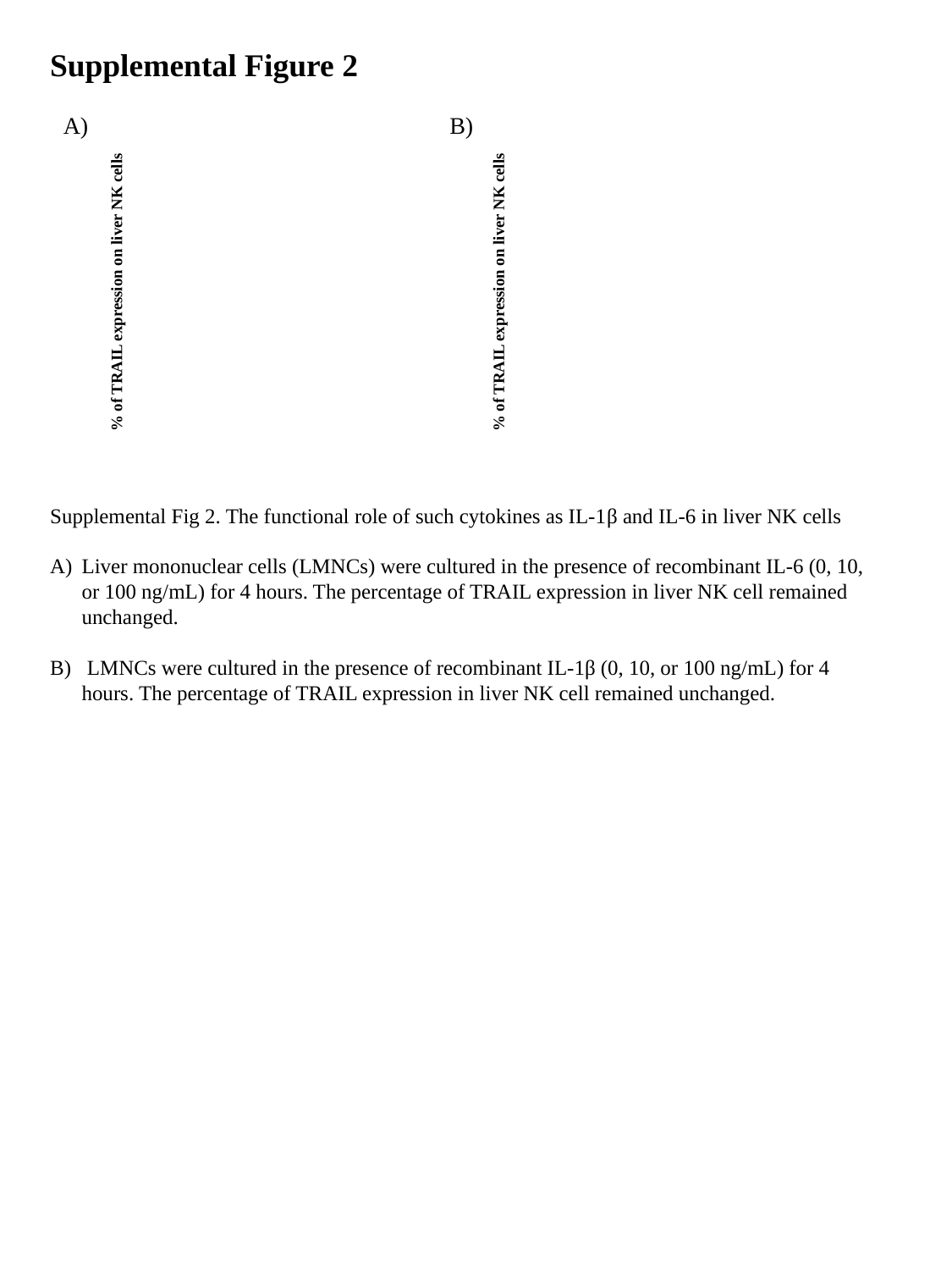

Supplemental Figure 2
A)
B)
% of TRAIL expression on liver NK cells
% of TRAIL expression on liver NK cells
Supplemental Fig 2. The functional role of such cytokines as IL‐1β and IL‐6 in liver NK cells
Liver mononuclear cells (LMNCs) were cultured in the presence of recombinant IL-6 (0, 10, or 100 ng/mL) for 4 hours. The percentage of TRAIL expression in liver NK cell remained unchanged.
 LMNCs were cultured in the presence of recombinant IL-1β (0, 10, or 100 ng/mL) for 4 hours. The percentage of TRAIL expression in liver NK cell remained unchanged.
