# Supplementary material for: Intestinal obstruction impairs the antitumor function of hepatic natural killer cells against colorectal cancer
Source: J Gastroenterol. 2026 Feb 6;61(4):450–61. doi: 10.1007/s00535-026-02349-w (PMC13048930; doi:10.1007/s00535-026-02349-w)
Supplement: Supplementary file 3 — Supplementary file3 Supplemental Table 1. Risk factors for lung metastasis. Supplemental Table 2. Risk factors for peritoneal dissemination. Supplemental Table 3. Risk factors for local recurrence (DOCX 57 KB) [file 535_2026_2349_MOESM3_ESM.docx]

Supplemental tables

Supplemental Table 1. Primers used for qPCR

| Gene | Forward | Reverse |
| --- | --- | --- |
| β2M | TGGTCTTTCTGGTGCTTGTC | GTATGTTCGGCTTCCCATTC |
| IL1β | CAACCAACAAGTGATATTCT | GATCCACACTCTCCAGCTGC |
| IL6 | CACAGAGGATACCACTCCCAACA | TCCACGATTTCCCAGAGAACA |
| TGFβ | TGACGTCACTGGAGTTGTACGG | GGTTCATGTCATGGATGGTGC |
| IL33 | GCTCACTGCAGGAAAGTACAG | TGGTCTTCTGTTGGGATCTTCTTA |

β2M, beta-2-microglobulin; IL1β, interleukin 1beta; IL6, Interleukin 6; TGFβ, transforming growth factor beta; IL33, Interleukin 33

Supplemental Table 2. Risk factors for lung metastasis

| Factors | Univariate | | | Multivariate | | |
| --- | --- | --- | --- | --- | --- | --- |
|  | HR | 95% CI | p-value | HR | 95% CI | p-value |
| Age, per 1 year | 1.00 | 0.99-1.02 | 0.669 |  |  |  |
| Gender (Male) | 1.02 | 0.73-1.44 | 0.895 |  |  |  |
| BMI (kg/m^2^), per 1 kg/m^2^ | 0.97 | 0.93-1.02 | 0.221 |  |  |  |
| PS |  |  |  |  |  |  |
| 0 | 1 |  |  |  |  |  |
| 1 | 1.10 | 0.72-1.67 | 0.670 |  |  |  |
| 2 | 1.48 | 0.81-2.70 | 0.205 |  |  |  |
| 3 | 0.98 | 0.24-3.99 | 0.980 |  |  |  |
| 4 | 2.05 | 0.29-14.7 | 0.980 |  |  |  |
| CCI, per 1 | 1.12 | 0.99-1.24 | 0.064 |  |  |  |
| CAR, per 1 | 0.82 | 0.60-1.01 | 0.068 |  |  |  |
| NLR, per 1 | 0.99 | 0.92-1.04 | 0.615 |  |  |  |
| CEA, per 1 ng/ml | 1.00 | 1.00-1.00 | 0.290 |  |  |  |
| CA19-9, per 1 U/ml | 1.00 | 1.00-1.00 | 0.963 |  |  |  |
| Tumor location (rectum) | 2.39 | 1.70-3.37 | < 0.01 | 2.36 | 1.65-3.37 | < 0.01 |
| Histological type (Por/muc) | 0.47 | 0.22-1.00 | 0.049 | 0.42 | 0.19-0.90 | 0.026 |
| pT4 | 1.62 | 1.12-2.35 | 0.014 | 1.56 | 1.05-2.30 | 0.026 |
| pN |  |  |  |  |  |  |
| N0 | 1 |  |  | 1 |  |  |
| N1 | 1.68 | 1.12-2.52 | 0.013 | 2.23 | 1.45-3.45 | < 0.01 |
| N2 | 3.00 | 1.85-4.87 | < 0.01 | 4.42 | 2.60-7.54 | < 0.01 |
| N3 | 7.94 | 4.19-15.0 | < 0.01 | 10.4 | 5.28-20.6 | < 0.01 |
| Obstruction (+) | 1.39 | 0.89-2.18 | 0.152 |  |  |  |
| No adjuvant chemotherapy | 1.38 | 0.97-1.95 | 0.071 | 2.58 | 1.76-3.79 | < 0.01 |
| Operation time, per 1min | 1.00 | 1.00-1.00 | 0.001 |  |  |  |
| Blood loss, per 1ml | 1.00 | 1.00-1.00 | 0.020 |  |  |  |

BMI, body mass index; NLR, neutrophil-to-lymphocyte ratio; CAR, C-reactive protein/albumin ratio; CCI, Charlson comorbidity index; PS, performance status; pT, pathological tumor; pN, pathological lymph node; Por/muc, poorly/mucinous; CEA, carcinoembryonic antigen; CA19-9, carbohydrate antigen 19-9

Supplemental Table 3. Risk factors for peritoneal dissemination

| Factors | Univariate | | | Multivariate | | |
| --- | --- | --- | --- | --- | --- | --- |
|  | HR | 95% CI | p-value | HR | 95% CI | p-value |
| Age, per 1 year | 1.03 | 1.01-1.05 | < 0.01 |  |  |  |
| Gender (Male) | 0.62 | 0.42-0.92 | 0.018 | 0.64 | 0.43-0.97 | 0.033 |
| BMI (kg/m^2^), per 1 kg/m^2^ | 0.96 | 0.91-1.01 | 0.105 |  |  |  |
| PS |  |  |  |  |  |  |
| 0 | 1 |  |  |  |  |  |
| 1 | 1.33 | 0.83-2.11 | 0.232 |  |  |  |
| 2 | 2.24 | 1.23-4.07 | < 0.01 |  |  |  |
| 3 | 2.05 | 0.64-6.55 | 0.224 |  |  |  |
| 4 | - | - | - |  |  |  |
| CCI, per 1 | 1.02 | 0.87-1.17 | 0.816 |  |  |  |
| CAR, per 1 | 1.08 | 0.94-1.20 | 0.233 |  |  |  |
| NLR, per 1 | 1.03 | 0.99-1.07 | 0.141 |  |  |  |
| CEA, per 1 ng/ml | 1.00 | 1.00-1.01 | 0.086 |  |  |  |
| CA19-9, per 1 U/ml | 1.00 | 1.00-1.00 | 0.198 |  |  |  |
| Tumor location (rectum) | 1.46 | 0.94-2.28 | 0.095 | 2.17 | 1.16-4.06 | 0.016 |
| Histological type (Por/muc) | 1.96 | 1.20-3.19 | < 0.01 | 2.42 | 1.22-4.83 | 0.012 |
| pT4 | 5.21 | 3.53-7.70 | < 0.01 | 4.28 | 2.82-6.49 | < 0.01 |
| pN |  |  |  |  |  |  |
| N0 | 1 |  |  |  |  |  |
| N1 | 1.50 | 0.96-2.35 | 0.077 | 1.61 | 0.99-2.57 | 0.052 |
| N2 | 3.05 | 1.82-5.12 | < 0.01 | 2.93 | 1.66-5.18 | < 0.01 |
| N3 | 3.82 | 1.50-9.72 | < 0.01 | 3.99 | 1.52-10.5 | < 0.01 |
| Obstruction (+) | 2.65 | 1.73-4.05 | < 0.01 | 2.04 | 1.32-3.15 | < 0.01 |
| No adjuvant chemotherapy | 1.26 | 0.85-1.86 | 0.257 | 1.87 | 1.23-2.84 | < 0.01 |
| Operation time, per 1min | 1.00 | 1.00-1.00 | 0.429 |  |  |  |
| Blood loss, per 1ml | 1.00 | 1.00-1.00 | 0.566 |  |  |  |

BMI, body mass index; NLR, neutrophil-to-lymphocyte ratio; CAR, C-reactive protein/albumin ratio; CCI, Charlson comorbidity index; PS, performance status; pT, pathological tumor; pN, pathological lymph node; Por/muc, poorly/mucinous; CEA, carcinoembryonic antigen; CA19-9, carbohydrate antigen 19-9

Supplemental Table 4. Risk factors for local recurrence

| Factors | Univariate | | | Multivariate | | |
| --- | --- | --- | --- | --- | --- | --- |
|  | HR | 95% CI | p-value | HR | 95% CI | p-value |
| Age, per 1 year | 0.99 | 0.97-1.01 | 0.617 |  |  |  |
| Gender (Male) | 1.55 | 0.86-2.80 | 0.142 |  |  |  |
| BMI (kg/m^2^), per 1 kg/m^2^ | 0.95 | 0.88-1.03 | 0.243 |  |  |  |
| PS |  |  |  |  |  |  |
| 0 | 1 |  |  |  |  |  |
| 1 | 1.11 | 0.54-2.28 | 0.766 |  |  |  |
| 2 | 1.86 | 0.72-4.79 | 0.201 |  |  |  |
| 3 | 1.50 | 0.20-11.0 | 0.691 |  |  |  |
| 4 | - | - | - |  |  |  |
| CCI, per 1 | 0.91 | 0.67-1.15 | 0.460 |  |  |  |
| CAR, per 1 | 1.18 | 1.01-1.31 | 0.039 |  |  |  |
| NLR, per 1 | 1.05 | 1.00-1.09 | 0.063 |  |  |  |
| CEA, per 1 ng/ml | 1.00 | 1.00-1.01 | 0.087 |  |  |  |
| CA19-9, per 1 U/ml | 1.00 | 1.00-1.00 | 0.829 |  |  |  |
| Tumor location (rectum) | 2.43 | 1.36-4.34 | 0.003 | 2.17 | 1.16-4.06 | 0.016 |
| Histological type (Por/muc) | 2.69 | 1.36-5.32 | 0.009 | 2.42 | 1.22-4.83 | 0.012 |
| pT4 | 4.46 | 2.49-7.98 | < 0.01 | 4.21 | 2.32-7.62 | < 0.01 |
| pN |  |  |  |  |  |  |
| N0 | 1 |  |  |  |  |  |
| N1 | 1.42 | 0.77-2.63 | 0.258 |  |  |  |
| N2 | 1.20 | 0.45-3.17 | 0.719 |  |  |  |
| N3 | - | - | - |  |  |  |
| Obstruction (+) | 1.42 | 0.66-3.05 | 0.366 |  |  |  |
| No adjuvant chemotherapy | 1.04 | 0.58-1.86 | 0.894 |  |  |  |
| Operation time, per 1min | 1.00 | 1.00-1.00 | < 0.01 | 1.004 | 1.001-1.006 | < 0.01 |
| Blood loss, per 1ml | 1.00 | 1.00-1.00 | < 0.01 |  |  |  |

BMI, body mass index; NLR, neutrophil-to-lymphocyte ratio; CAR, C-reactive protein/albumin ratio; CCI, Charlson comorbidity index; PS, performance status; pT, pathological tumor; pN, pathological lymph node; Por/muc, poorly/mucinous; CEA, carcinoembryonic antigen; CA19-9, carbohydrate antigen 19-9
